# The Matrons' Corner

**Published:** 1887-12-10

**Authors:** R. Norris


### The Matrons' Corner.

### "LADY COOKS FOR HOSPITALS."

Why do not those who advocate the plan just put it into practice? It has been tried in one of the largest hospitals in the kingdom with the happiest results. The first necessity is of course "to catch your cook," who must of necessity have a talent for cooking; must next have the qualities that make a good mistress of a household, and must not less have the good sense and dignity which will enable her to grapple with the difficulties of the situation, and to overcome them quietly. Roman Catholics set the example in their organisations. With them one finds a Sister in the kitchen, one over the laundry, and one over the linenry—just as over a hospital ward, or school, or any other department of their work; and this without any parade of condescension. In the same hospital a like plan has been adopted in the laundry and linenry, a Sister being placed over each department. Such experiments should be made quietly, and need not be discussed beforehand. R. Norris.

